# Journal of Neurodevelopmental Disorders reviewer acknowledgement 2015

**DOI:** 10.1186/s11689-016-9137-x

**Published:** 2016-02-10

**Authors:** Joseph Piven

**Affiliations:** Carolina Institute for Developmental Disabilities, University of North Carolina at Chapel Hill, 100 Renee Lynne Court, Carrboro, NC 27510 USA

## Abstract

We thank all of the reviewers who have contributed to the journal in volume 7 (2015). High quality and timely reviews are critical to the overall quality of the journal. We are committed to providing a unique and important outlet for scholarship regarding neurodevelopmental disorders and are indebted to the outstanding reviewers who have contributed their time over the last year in helping us to reach this goal.

**Ted Abel**

United States of America

**Kevin Antshel**

United States of America

**Simon Baron-Cohen**

United Kingdom

**Carrie Bearden**

United States of America

**Nan Bernstein Ratner**

United States of America

**Thomas Bourgeron**

France

**Randal Carpenter**

United States of America

**Tony Charman**

United Kingdom

**Katarzyna Chawarska**

United States of America

**Rina Cianfaglione**

United Kingdom

**Claire Coles**

United States of America

**John Colombo**

United States of America

**John Constantino**

United States of America

**Cheryl Corcoran**

United States of America

**Jacqueline Crawley**

United States of America

**Julie Daniels**

United States of America

**Petrus de Vries**

South Africa

**William DeBassio**

United States of America

**Adele Dimian**

United States of America

**Anastasia Dimitropoulos**

United States of America

**Ilan Dinstein**

Israel

**Dennis Drayna**

United States of America

**Inge-Marie Eigsti**

United States of America

**Jed Elison**

United States of America

**Susan Faja**

United States of America

**Reinhold Feldmann**

Germany

**Susanna Fryer**

United States of America

**Oscar Gershanik**

Argentina

**Simon Gregory**

United States of America

**Amanda Hampton Wray**

United States of America

**Pamela Heaton**

United Kingdom

**Melissa Hines**

United Kingdom

**Lisa M Jacola**

United States of America

**Anna Jansen**

Belgium

**Chris Jarrold**

United Kingdom

**Loydie Jerome-Majewska**

Canada

**Shafali Jeste**

United States of America

**Tracy Jirikowic**

United States of America

**Emily Jones**

United Kingdom

**Robert Joseph**

United States of America

**Matthew Judson**

United States of America

**Annette Karmiloff-Smith**

United Kingdom

**Wendy Kates**

United States of America

**Brandon Keehn**

United States of America

**Kelly King**

United States of America

**Bonita Klein-Tasman**

United States of America

**Ami Klin**

United States of America

**Rebecca Knickmeyer**

United States of America

**Alexander Kolevzon**

United States of America

**Barry Kosofsky**

United States of America

**Rebecca Landa**

United States of America

**Janine LaSalle**

United States of America

**Catherine Lebel**

Canada

**Eva Loth**

United Kingdom

**Rebecca Lucas**

United Kingdom

**Bryan Luikart**

United States of America

**Suzanne Macari**

United States of America

**Marsha Mailick**

United States of America

**Christian Marshall**

Canada

**Klara Marton**

United States of America

**Joe McCleery**

United States of America

**Jennifer Miller**

United States of America

**Eric Morrow**

United States of America

**Matt Mosconi**

United States of America

**Jeff Munson**

United States of America

**Michael Murias**

United States of America

**Clodagh Murphy**

United Kingdom

**Charles Nelson**

United States of America

**Rowena Ng**

United States of America

**Susan Nittrouer**

United States of America

**Courtenay Norbury**

United Kingdom

**Christine Nordahl**

United States of America

**Lindsay Oberman**

United States of America

**Lucy Osborne**

Canada

**Noriko Osumi**

Japan

**Opal Ousley**

United States of America

**David Perrett**

United Kingdom

**Sarika Peters**

United States of America

**Daniela Plesa Skwerer**

United States of America

**Elizabeth Powell**

United States of America

**Natalie Pride**

Australia

**Zhenghan Qi**

United States of America

**Andrea Quintero**

United States of America

**Peter Rabins**

United States of America

**Armin Raznahan**

United States of America

**Avi Reichenberg**

United States of America

**Leslie Rescorla**

United States of America

**Elisabeth Roof**

United States of America

**Joanne Rovet**

Canada

**Daniela Rubin**

United States of America

**Mustafa Sahin**

United States of America

**Mark Sands**

United States of America

**Michael Schmeisser**

Germany

**Elliott Sherr**

United States of America

**Frederick Shic**

United States of America

**Linmarie Sikich**

United States of America

**Tony Simon**

United States of America

**Mikle South**

United States of America

**Sarah Spence**

United States of America

**Dimitri Stavropoulos**

Canada

**Kyle Steinman**

United States of America

**Andre Strydom**

United Kingdom

**John Sweeney**

United States of America

**Yukari Takarae**

United States of America

**Kristiina Tammimies**

Sweden

**Raffaella Tancredi**

Italy

**Amanda Tarullo**

United States of America

**Marc Tasse**

United States of America

**Lucina Q Uddin**

United States of America

**Andrea Vaags**

Canada

**Therese van Amelsvoort**

Netherlands

**Giacomo Vivanti**

United States of America

**Sam Wass**

United Kingdom

**Jason Wolff**

United States of America

**Kate Woodcock**

United Kingdom

**Gregory Young**

United States of America

**Richard Ziegler**

United States of America

